# mtDNA CR Evidence Indicates High Genetic Diversity of Captive Forest Musk Deer in Shaanxi Province, China

**DOI:** 10.3390/ani13132191

**Published:** 2023-07-04

**Authors:** Zhe Wang, Guanjie Lu, Yunyun Gao, Liping Yan, Mingzhe Li, Defu Hu, Dong Zhang

**Affiliations:** 1 School of Ecology and Nature Conservation, Beijing Forestry University, Qinghua East Road 35, Beijing 100083, China; wzhe0314@163.com (Z.W.); luguanjie2023@163.com (G.L.); en130434@bjfu.edu.cn (Y.G.); yanlp@bjfu.edu.cn (L.Y.); hudf@bjfu.edu.cn (D.H.); 2 China Wildlife Conservation Association, Beijing 100714, China; limingzhe92@126.com

**Keywords:** forest musk deer, mtDNA control region, genetic diversity, haplotype, maternal line

## Abstract

**Simple Summary:**

Forest musk deer (*Moschus berezovskii*) is a species that is currently classified as endangered and found in China and Vietnam. In order to prevent their extinction, China initiated captive breeding programs in the 1950s. Maintaining the high genetic diversity of the population is a crucial factor for ensuring the sustainable and rapid growth of these captive populations. Mitochondrial DNA (mtDNA) possesses a unique lineage of matrilineal evolution, and the control region (CR) is considered to be one of the most popular molecular markers for conducting a genetic diversity analysis and determining maternal lines. In this study, we assessed the current genetic diversity status of 338 individuals from seven captive forest musk deer populations located in the Shaanxi province using mtDNA CR, and the results showed that the genetic diversity was high. We also made full use of previous mtDNA CR data clearly defining 65 haplotypes with their frequency and concluded with about 90 maternal lines. The analysis revealed no significant genetic differentiation among the populations, and the populations might not have experienced rapid population expansion. The current study represents the most comprehensive research of genetic diversity of captive forest musk deer to date, and will be helpful to preserve and enhance the genetic diversity of the captive forest musk deer populations.

**Abstract:**

Forest musk deer (*Moschus berezovskii*) are endangered ruminants whose adult males secrete musk. China has been breeding forest musk deer artificially since the 1950s in an effort to restore wild populations, with Shaanxi and Sichuan provinces as the two main sites for captive breeding. Genetic diversity is a significant indicator that determines the long-term viability and status of a population, particularly for species at risk of extinction. In this study, we analyzed the current genetic makeup of seven captive forest musk deer populations in the Shaanxi province, using the mitochondrial DNA (mtDNA) control region (CR) as the molecular marker. We sequenced 604 bp of mtDNA CR, with an average content of A+T higher than G+C. We observed 111 variable sites and 39 different haplotypes from 338 sequences. The nucleotide diversity (Pi) and haplotype diversity (Hd) were 0.02887 and 0.908, respectively. Genetic differentiation between these populations was not significant, and the populations might not have experienced rapid growth. By combining our sequences with previous ones, we identified 65 unique haplotypes with 26 rare haplotypes and estimated a total of 90 haplotypes in Shaanxi province captive populations. The Shaanxi province and Sichuan province obtained 88 haplotypes, the haplotypes from the two populations were mixed together, and the two populations showed moderate genetic differentiation. Our findings suggested that captive forest musk deer populations in the Shaanxi province had high genetic diversity, with a rich founder population of about 90 maternal lines. Additionally, managers could develop genetic management plans for forest musk deer based on the haplotype database. Overall, our study will provide insights and guidelines for the conservation of genetic diversity in captive forest musk deer populations in the Shaanxi province.

## 1. Introduction

The process of genetic diversity ensues through the mutation and aggregation of genetic material during the evolution of a species [1]. As the genetic diversity of a species increases, its capacity for evolutionary potential is amplified, thereby augmenting its ability to cope with changes in the environment [2,3]. To successfully preserve an endangered species, it is essential to not only shield its population but also to conserve its genetic diversity for the prospects of its long-term survival [4,5].

Musk deer (*Moschus* spp.), indigenous to East Asia with solitary nature and high stress, are renowned for the secretion of musk by adult males. At one point, China housed the largest population of musk deer, accounting for 70% of the total population with an estimated 2.5 million wild musk deer in the 1950s [6]. Miserably, the musk deer population has dwindled significantly as their existence has been threatened due to human encroachment on their habitat and other contributing factors. Currently, they are found only in a few areas, hovering close to extinction [7].

The forest musk deer (*Moschus berezovskii*), one of seven musk deer species and the most widespread in China, continues to face a perilous situation, with its population listed as Endangered on the IUCN Red List [8]. To prevent the extinction of these wild populations, the Chinese government, in 1958, authoritied to commence captive breeding of musk deer in Shaanxi, Sichuan, and Anhui provinces. Over the last 60 years, China has made some strides in the artificial breeding of forest musk deer, with the total number of captive forest musk deer exceeding 30,000 [9]. However, a major concern persists as the lineage of most captive forest musk deer is ambiguous, lacking strict documentation. Inbreeding in such populations often results in a decrease in genetic diversity and a higher risk of disease. Therefore, it is paramount to assess the genetic status of captive populations and try to trace the pedigree to conserve the forest musk deer resources.

The advent of molecular marker technology has provided an effective tool for conserving the genetic diversity of species [10,11]. Mitochondrial DNA (mtDNA), the genetic material outside the nucleus, is characterized by maternal transmission and a faster rate of evolution than nuclear DNA (nDNA) [12,13,14]. In particular, its control region (CR), which has a main function related to mtDNA replication, termination, and transcription, as a non-coding region, does not participate in protein coding, so it is subjected to the least selection pressure and the fastest evolution speed [15]. mtDNA CR is generally used for the research of intra-species differentiation and is a useful molecular marker for the study of genetic diversity [16]. Alvarez et al. [17] found that the genetic background established by mtDNA CR was comparable to that in the studbooks. mtDNA CR has also been demonstrated in numerous articles to be an effective tool for maternal line reconstruction [18] or was used for the correction of dam lines [19].

Previous studies on the genetic diversity of forest musk deer based on mtDNA CR markers only focused on the comparison of the genetic diversity of various groups. Here, we introduced Chao 1, which is used to estimate richness in ecological studies, to estimate the number of all haplotypes. This research level included assessing the current genetic diversity as well as tracing the maternal lines of the captive forest musk deer in the Shaanxi province.

## 2. Materials and Methods

### 2.1. Sample Collection

We obtained feces, tissue, and blood samples from a total of 338 forest musk deer in seven captive populations: randomly sampled from Feng County (35 different farms; R, *n* = 35), Fumin (medium farm: more than 100 individuals; FM, *n* = 46), Pianzaihuang (large farm: more than 200 individuals; PZH, *n* = 44), Haixing (large farm; HX, *n* = 118), Liangdang (small farm: with fewer than 100 individuals; LD, *n* = 39), Hongda (medium farm; HD, *n* = 30), and Guanling (medium farm; GL, *n* = 26). Except for LD, which is situated in the Gansu province but where the forest musk deer population has been introduced from the Shaanxi province, all other populations are located in the Shaanxi province. In the process of sampling, we adhered to the principle of random sampling and did not know the genetic relationship of each population. We stored the samples in a laboratory refrigerator at a temperature of −20 °C until DNA extraction.

### 2.2. DNA Extraction and Amplification

The method reported by Tang et al. [20] was used to process fecal samples into cell suspensions. Subsequently, genomic DNA was extracted utilizing the DNeasy Blood/Tissue/Cell Kit (TIANGEN) and stored at −80 °C for future use.

The mtDNA CR was amplified by a polymerase chain reaction (PCR) using the primers F: 5′-CAACTAACCTCCCTAAGACTTCAAG-3′ and R: 5′-CCAAATGTATGACAGCACAGTTATG-3′. The PCR amplification volume was 25 μL, including 12.5 μL 2 × Taq PCR Master Mix (Dye), 30–50 ng DNA template, 1 μL forward primer, and 1 μL reverse primer. The conditions were performed as follows: initial denaturation at 94 °C for 5 min, followed by 34 cycles of PCR (denaturation at 94 °C for 30 s, annealing at 57 °C for 30 s, and extension at 72 °C for 1 min), and a final extension of 72 °C for 8 min. PCR products were examined by electrophoresis on a 1.0% agarose gel. All reagents and labware used are listed in Tables S1 and S2, respectively. PCR products with bands detected by electrophoresis on a 1.0% agarose gel were sent to The Beijing Genomics Institute for purification and Sanger sequencing bidirectionally.

### 2.3. Data Analysis

Individual mtDNA CR sequences were edited by the SeqMan Pro program in DNASTAR Lasergene version 7.1 (DNASTAR Inc., Madison WI, USA) [21] and then rechecked manually. Molecular Evolutionary Genetics Analysis version 5 (MEGA 5, Mega Limited, Auckland, New Zealand) [22] was used to align all sequences, determine the nucleotide composition, calculate the genetic distance using the Kimura 2-parameter model, and then construct a neighbor-joining (NJ) phylogenetic tree with 1000 bootstrap replicates. The variable sites, number of haplotypes, nucleotide diversity (Pi), haplotype diversity (Hd), average number of nucleotide differences (K), and mismatch distribution were determined for each population by DNA Sequence Polymorphism version 6 (DnaSP 6, Julio Rozas’ Research Group · Universitat de Barcelona, Barcelona, Spain) [23]. Statistical Estimation of Species Richness and Shared Species from Samples version 9.1.0 (EstimateS 9.1.0, Professor Robert K. Colwell, Colorado, America) [24] was applied to estimate the total haplotype richness based on the classic Chao 1 Richness Estimator (Chao-1); its 95% confidence intervals (95% CIs) were computed using 1000 permutations. A median-joining network was constructed using Population Analysis with Reticulate Trees version 1.7 (POPART 1.7, Dr. Jessica W. leigh, Dunedin, New Zealand) [25]. Genetic differentiation (*Fst*), analysis of molecular variance (AMOVA), Tajima’s *D* (reflects long-term population events), and Fu’s *Fs* (relatively sensitive to recent population events) statistics were carried out with Arlequin 3.5 (Swiss Institute of Bioinformatics, Lausanne, Switzerland) [26]. The level of significance was set at *p* < 0.05; the extremely significant level was set at *p* < 0.01; *p* > 0.05 indicates no significant difference.

### 2.4. Data Acquisition of mtDNA CR

In order to build the existing haplotype frequency of captive forest musk deer in the Shaanxi province and infer all haplotypes, mtDNA CR sequences from captive forest musk deer in the Shaanxi province were downloaded from NCBI, accession: JX499255-69 [27], MH047347 [28], OM100019-54 [29], KR074194-197, KR074199, KR074202, KR074205, and KR074207 [30]; in addition, seven mtDNA CR haplotype sequences were obtained in Guo’s [31] master’s thesis, and the number of occurrences for each haplotype was determined based on relevant articles. In order to find out whether the Shaanxi population and the Sichuan population are differentiated, so as to provide the basis for the subsequent provenance exchange, 112 mtDNA CR sequences from captive forest musk deer in the Sichuan province were also obtained from NCBI, accession: EU795773-881 [32], KR074200 [30], JQ409122, and MW879208.

## 3. Results

### 3.1. Genetic Diversity

**Figure 1:**
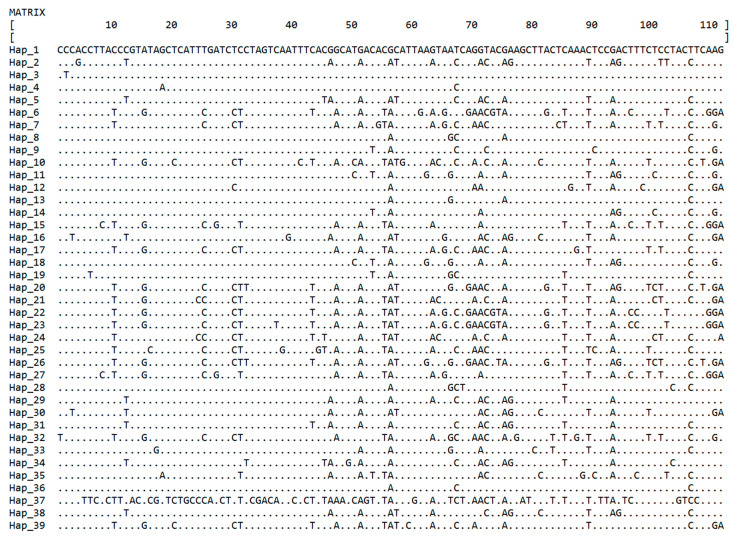
The variation sites of 39 mtDNA CR haplotypes of captive forest musk deer in Shaanxi province.

Upon editing and multiple sequence alignment, the effective sequence length of mtDNA CR for 338 forest musk deer was 604 bp. The nucleotide composition was typically A+T-biased, with an average content of A+T (63.3%) higher than that of G+C (36.7%). The total number of sites, excluding those with gaps or missing data, was 597, with 7 sites containing alignment gaps or missing data. Invariable sites were 486, variable sites were 111, and the total number of mutations was 114. Singleton variable sites with two variants were 5, while parsimony informative sites with two variants were 103. There were no singleton variable sites with three variants, but there were 3 parsimony informative sites with three variants. Variable sites with four variants were absent. The Pi was 0.02887, and the K was 17.236. In total, 39 haplotypes were defined for the 338 individuals, and the Hd was 0.908. The HD population had the highest Pi, while the LD population had the lowest Pi. PZH had the highest Hd, whereas HX had the lowest Hd. There was no significant difference between Pi and HD in various populations. The information regarding haplotype variation sites is illustrated in Figure 1, while the genetic information for the seven populations is presented in Table 1.

### 3.2. Haplotype Analyses

We obtained a total of 65 known haplotypes, and the total haplotype richness at the sampling site, estimated using the sample-based Chao-1 estimator, is 90 (95% CI: 75–130). The range of genetic distance, according to the Kimura 2-parameter model, between each haplotype was 0.002 to 0.131, and the average genetic distance was 0.041. Table 2 shows the frequency and distribution of haplotypes. Hap8 was the most frequent haplotype, shared by 92 individuals out of 553, accounting for approximately 16.64%. Hap3 was the second most frequent haplotype, accounting for 12.48% of all individuals. In total, 26 haplotypes appeared only once and were deemed rare haplotypes. Haplotype distribution was extremely uneven in each population, with FM having individuals sharing Hap3 and Hap12, each accounting for over 30% of the population; in HX, Hap23 had a frequency of more than 20%, while Hap8 had a frequency of more than 40%; in LD, Hap3 and Hap4 accounted for more than 30%, respectively, of the total; Hap8 accounted for more than 30% each of the HD population and the GL population. The proportion of each haplotype in R and PZH was relatively balanced, without too many single haplotypes. Hap5 was exclusive to R, Hap11 and Hap13 (appeared twice) were exclusive to FM, Hap17, Hap27, and Hap28 were exclusive to PZH, and Hap34 and Hap36 were exclusive to LD.

**Figure 2:**
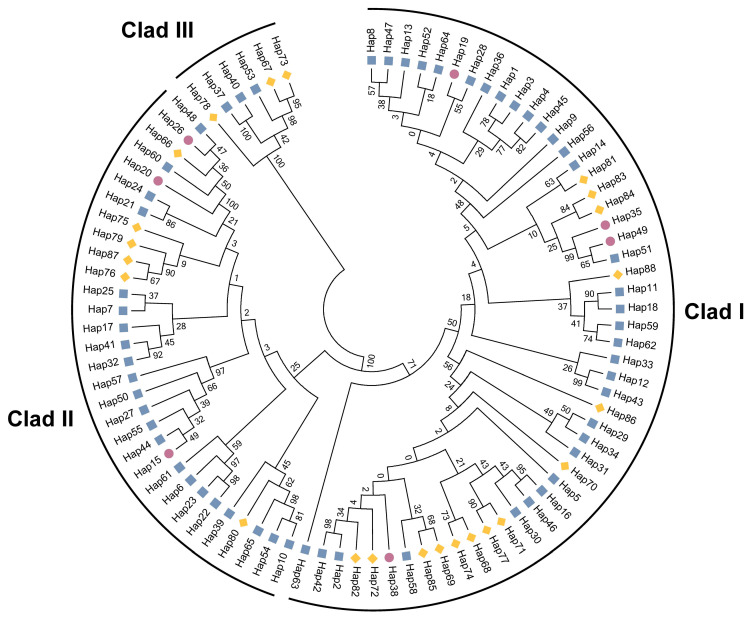
The neighbor-joining phylogenetic tree of mtDNA control region haplotypes of captive forest musk deer in Shaanxi population and Sichuan population. Note: Blue: Shaanxi; yellow: Sichuan; purple: shared.

A total of 88 haplotypes of forest musk deer were obtained by combining the existing haplotypes from Shaanxi and Sichuan provinces, and 7 haplotypes were shared between the two populations. Based on genetic distance, we constructed an NJ tree with 1000 bootstrap replicates (Figure 2). All haplotypes were classified into three distinct clades, each of which varied greatly in size and included rare haplotypes. Except for the shared haplotype, the haplotypes from the Shaanxi population and Sichuan population were not separated. Additionally, Clade Ⅰ had the largest number of haplotypes and was considered the dominant haplogroup, followed by Clade Ⅱ and then Clade Ⅲ. The median-joining network clustering results were consistent with those of the NJ tree (Figure S1).

### 3.3. Genetic Structure

Data from this study and the Sichuan population were used in the genetic structure study, and Table 3 shows the mean genetic distances between and within each population. The genetic distance was highest between HD and PZH, as well as HD and HX, and lowest between GL and LD. The maximum genetic distance was observed within HD, while the minimum was within LD. It was apparent that the genetic distance between HD and the other populations was large. The genetic distance between the Shaanxi and Sichuan populations was greater than that within the Shaanxi population and less than that within the Sichuan population.

Table 4 shows the *Fst* between each of the populations and *p*-values. The analysis results display that HX and FM, as well as HX and LD, were highly differentiated, with the *Fst* value of more than 0.15. Most of the populations showed moderate differentiation from each other, while a few populations exhibited low differentiation. Specifically, the Shaanxi and Sichuan populations demonstrated moderate differentiation from each other.

The AMOVA involved the separation of variation between populations (Va) and within populations (Vb), followed by a significance test (Table 5). The results show that 9.47% of the total variation was attributed to variation among populations, while 90.53% was attributed to variation within populations. The fixation index was 0.09468, indicating a highly significant difference. These results suggest that the majority of genetic variation in the population was from within the population itself.

### 3.4. Demographic History

**Figure 3:**
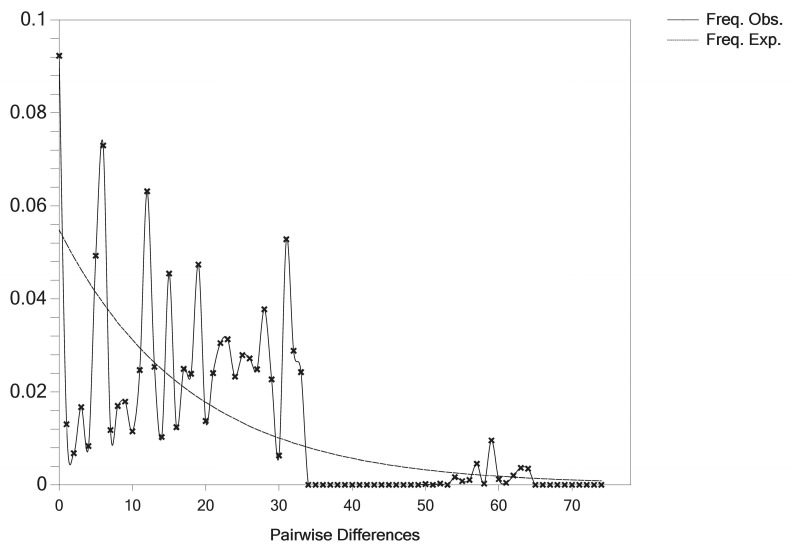
Observed (solid lines) and expected (dotted lines) mismatch distribution for captive forest musk deer populations in Shaanxi province.

Regarding the results of the neutrality tests, Tajima’s *D* and Fu’s *Fs* did not achieve statistical significance (Table S3), indicating that the captive forest musk deer population in the Shaanxi province did not depart from the neutral selection. The mismatch distribution curve shows a multi-peak distribution (Figure 3), which does not adhere to the model of population expansion. These suggest that none of the populations might have undergone rapid population expansion.

## 4. Discussion

Captive breeding followed by reintroduction is an indispensable technique to safeguard endangered species [33,34]. This methodology has been successfully implemented in preserving the Przewalski horse (*Equus ferus*) [35], Persian fallow deer (*Dama mesopotamica*) [36], and Pere David’s deer (*Elaphurus davidianus*) [37]. Its purpose is not only to rapidly increase the population but also to avoid inbreeding depression and ensure genetic variability over the long term [34]. Understanding the genetic status of captive forest musk deer populations will help us better protect their genetic diversity [38,39].

This was the largest sampling range research on the genetic diversity of captive forest musk deer populations in the Shaanxi province, aiming to objectively evaluate genetic diversity and the total number of haplotypes. Pi is defined as the average number of nucleotide differences at each site between randomly selected DNA sequences in a given population. Hd refers to the frequency at which two different haplotypes are randomly selected from a sample. Pi and Hd are commonly used to measure genetic diversity, with higher values indicating a greater polymorphism in the population and a more abundant genetic diversity [40]. The Pi and Hd of 338 forest musk deer in seven populations were 0.02887 and 0.908, respectively. Compared to previous studies, the Pi and Hd values were lower than those found in captive [29] and wild [41] forest musk deer populations in the Shaanxi province, as well as in Sichuan province captive forest musk deer populations [32], but higher than those of wild forest musk deer in the Sichuan province [42]. According to the study of Was and Bowen [43], a higher Pi than 0.005 and a higher Hd than 0.5 indicated a higher genetic diversity in the population. The results of this study were all higher than the standard value, which means that forest musk deer still exhibited high genetic diversity. We inferred with Chao 1 that there were 90 mtDNA CR haplotypes of captive forest musk deer in the Shaanxi province, including 90 maternal lines [44]. The reasons for the high genetic diversity of forest musk deer may be the abundant maternal lines with rich genetic resources and short breeding time. It is crucial to pay attention to the conservation of their genetic resources. For the seven populations, the ordering of Pi and Hd was not consistent. This likely relates to the increase in population number, leading to the accumulation of haplotype diversity through mutation, but insufficient time for the diversity of nucleotide sequences to accumulate [43].

We conducted the analysis of the frequency of known haplotypes; of the 65 haplotypes, 26 haplotypes were rare haplotypes. In populations with high genetic diversity, rare haplotypes are at risk of being lost when the carrier dies. Therefore, increasing the frequency of rare haplotypes in the population is crucial. One noteworthy issue is that multiple individuals share the same haplotype, except for R and PZH populations, on account of an unbalanced representation of the haplotypes affecting the genetic diversity [28]. The forest musk deer in Feng County were all classified as the R population, while the forest musk deer of population PZH were collected from multiple farms with a wide range of sources, so none of these problems occurred.

The genetic structure analysis indicated that high genetic variation exists within populations, but low genetic differentiation exists between populations [45]. With little genetic differentiation between captive populations, they can be adjusted based on the haplotype distribution of each musk deer farm. Moreover, the issue of disease transmission between different populations should be considered. According to our study and the article published by Liu et al. [46], there was no significant genetic differentiation in Shaanxi and Sichuan populations. The haplotypes of the two populations are mixed together, and they have seven shared haplotypes. However, whether to switch the provenance remains a topic for future investigation.

Tajima [47] believed that if both Tajima’s *D* and Fu’s *Fs* values are negative and the difference is statistically significant (*p* < 0.05), the sequence contains more change in nucleotide sites than the neutral evolutionary model, which might indicate that the population has experienced a history of expansion. With a nucleotide mismatch analysis, the mismatch distribution curve had a single peak, which accords with the theoretical distribution expected by the population expansion model [26]. In this study, Tajima’s *D* and Fu’s *Fs* did not reach statistical significance, and the mismatch distribution curve presented multiple peaks; this meant that there was no significant population expansion of forest musk deer in the Shaanxi province. This may have something to do with the habits and reproduction of forest musk deer. Forest musk deer have strong territorial behavior and are highly stressed [48]. Even after 60 years of captivity, they still maintain their original habits. Additionally, they breed once a year and give birth to one to three young per birth, mostly two. All these prevent forest musk deer from expanding in large numbers, even in captivity and conservation.

## 5. Conclusions

To sum up, our research aimed to evaluate the genetic diversity of seven captive forest musk deer populations in the Shaanxi province and trace maternal lines. Our findings showed that the genetic diversity was abundant and the differentiation among each population was not very high. Furthermore, the populations did not undergo a fast population expansion. We also identified about 90 maternal lines and created a haplotype database. We firmly believe that our study will aid the breeding and conservation of captive forest musk deer in the Shaanxi province.

## Figures and Tables

**Table 1:** Genetic diversity parameters of seven captive forest musk deer populations in Shaanxi province.

Population	Sample Size (n)	Number of Variable Sites	Number of Haplotypes (H)	Nucleotide Diversity (Pi)	Haplotype Diversity (Hd)	Average Number of Nucleotide Differences (k)
R	35	54	10	0.02583	0.892	15.499
FM	46	47	9	0.01925	0.794	11.553
PZH	44	70	24	0.02686	0.959	18.462
HX	118	61	14	0.02794	0.764	16.763
LD	39	56	14	0.01816	0.854	10.880
HD	30	84	7	0.04385	0.811	26.223
GL	26	51	12	0.01901	0.886	11.403
Total	338	111	39	0.02887	0.908	17.236

**Table 2:** Distribution of mtDNA CR haplotype of captive forest musk deer populations in Shaanxi province.

		Population	R	FM	PZH	HX	LD	HD	GL	Published	Total
	Frequency										
Haplotype											
Hap1			5		5	3	1			11	25
Hap2			6	4	6	2	6	5	1	8	38
Hap3			5	15	2	5	9	2	2	29	69
Hap4			6		1	1	11		3	13	35
Hap5			1								1
Hap6			2			5				12	19
Hap7			1							2	3
Hap8			3		2	49	2	11	8	17	92
Hap9			1	3	2	1	2		2	1	12
Hap10			5		3	3		3		7	21
Hap11				1							1
Hap12				14	3	1	1	3	1	2	25
Hap13				2							2
Hap14				2	1					2	5
Hap15				4						13	17
Hap16				1	1		1			9	12
Hap17					1						1
Hap18					1					1	2
Hap19					2				2	5	9
Hap20					1				1	1	3
Hap21					3					2	5
Hap22					1					1	2
Hap23					1	28				5	34
Hap24					1		1	1		3	6
Hap25					1		1				2
Hap26					2					2	4
Hap27					1						1
Hap28					1						1
Hap29					1	4			3	14	22
Hap30					1					1	2
Hap31						4				4	8
Hap32						8			1		9
Hap33						4	1			2	7
Hap34							1				1
Hap35							1			1	2
Hap36							1				1
Hap37								5		2	7
Hap38									1	1	2
Hap39									1	1	2
Hap40										5	5
Hap41										1	1
Hap42										1	1
Hap43										1	1
Hap44										1	1
Hap45										1	1
Hap46										2	2
Hap47										1	1
Hap48										2	2
Hap49										8	8
Hap50										1	1
Hap51										1	1
Hap52										1	1
Hap53										1	1
Hap54										1	1
Hap55										1	1
Hap56										2	2
Hap57										1	1
Hap58										1	1
Hap59										2	2
Hap60										1	1
Hap61										1	1
Hap62										1	1
Hap63										1	1
Hap64										1	1
Hap65										3	3

**Table 3:** The mean genetic distances between (under the diagonal) and within (on the diagonal) seven captive forest musk deer populations in Shaanxi province, as well as Shaanxi and Sichuan populations.

Population	R	FM	PZH	HX	LD	HD	GL	Shaanxi	Sichuan
R	0.027								
FM	0.025	0.020							
PZH	0.030	0.028	0.032						
HX	0.031	0.030	0.032	0.029					
LD	0.023	0.021	0.029	0.029	0.019				
HD	0.040	0.039	0.042	0.042	0.038	0.047			
GL	0.024	0.022	0.028	0.027	0.020	0.036	0.020		
Shaanxi								0.032	
Sichuan								0.045	0.050

**Table 4:** The genetic differentiation between each of the populations (under the diagonal) and p-values (above the diagonal).

Population	R	FM	PZH	HX	LD	HD	GL	Shaanxi	Sichuan
R		**0.00000**	0.12613 ±0.0309	**0.00000**	0.08108 ±0.0286	**0.01802** **±0.0182**	0.12613 ±0.0364		
FM	0.08076		**0.00000**	**0.00000**	**0.00000**	**0.00000**	**0.00000**		
PZH	0.02023	0.08415		**0.00000**	**0.00000**	**0.00000**	**0.00901** **±0.0091**		
HX	0.07689	0.16246	0.05156		**0.00000**	**0.00000**	**0.00000**		
LD	0.02444	0.08726	0.10643	0.15678		**0.00000**	**0.02703** **±0.0194**		
HD	0.06420	0.14706	0.05357	0.09365	0.13224		**0.02703** **±0.0194**		
GL	0.02497	0.10263	0.06311	0.08097	0.03319	0.06902			
Shaanxi									**0.00000**
Sichuan								0.10404	

Note: Bold font means *p*-values < 0.05.

**Table 5:** AMOVA of captive forest musk deer populations in Shaanxi province.

Source of Variation	Degrees of Freedom	Sum of Squares	Variance Components	Percentage of Variation
Among populations	6	273.781	0.83116 Va	9.47
Within populations	331	2630.452	7.94699 Vb	90.53
Total	337	2904.234	8.77814	
Fixation Index	0.09468 (++)			

Note: (++) means *p*-value < 0.01.

## Data Availability

The mtDNA CR haplotype sequence data are available from the NCBI, accession: OQ718515—OQ718553.

## Supplementary Materials

The following supporting information can be downloaded at: https://www.mdpi.com/article/10.3390/ani13132191/s1, Figure S1: The median-joining network of mtDNA control region haplotypes of captive forest musk deer in Shaanxi population and Sichuan population; Table S1: The information of reagents used; Table S2: The information of labware used; Table S3: Tajima’s *D* and Fu’s *Fs* tests of captive forest musk deer populations in Shaanxi Province.
