# Supplementary material for: mtDNA CR Evidence Indicates High Genetic Diversity of Captive Forest Musk Deer in Shaanxi Province, China
Source: Animals (Basel). 2023 Jul 4;13(13):2191. doi: 10.3390/ani13132191 (PMC10339889; doi:10.3390/ani13132191)
Supplement: Supplementary file 1 [file animals-13-02191-s001.zip › Figure S1. The median-joining network of mtDNA control region haplotypes of captive forest musk deer in Shaanxi population and Sichuan population..pdf]

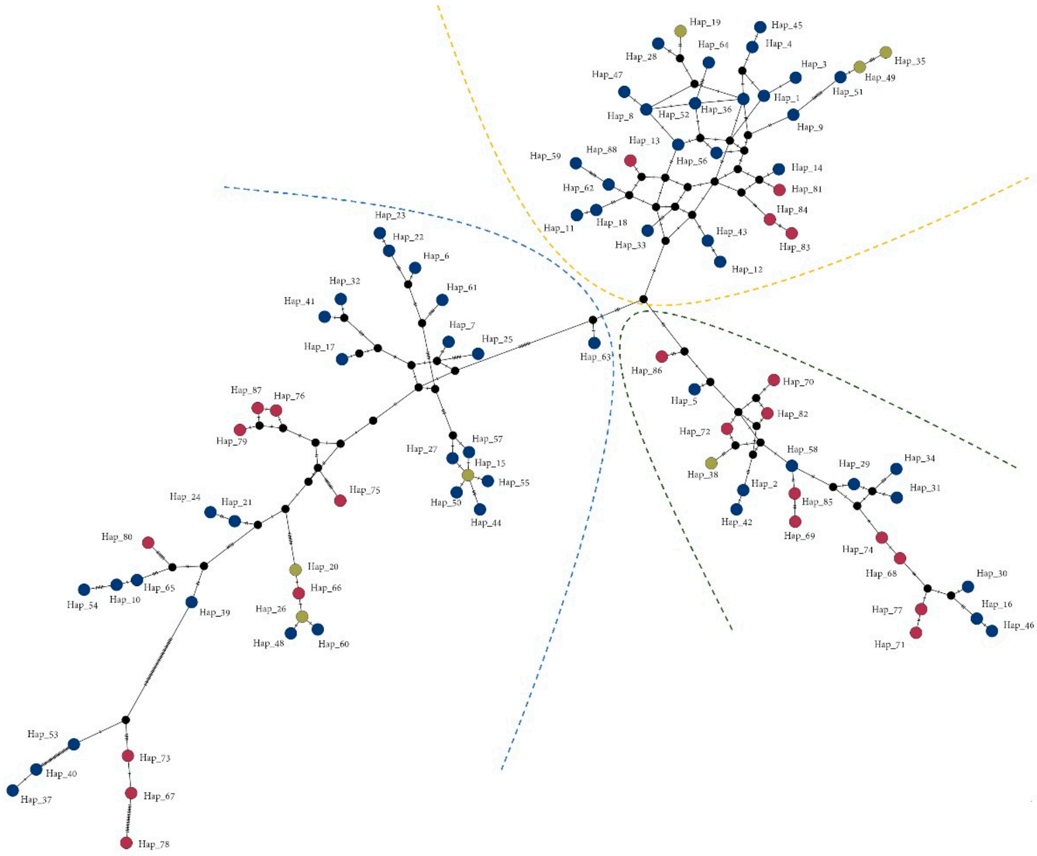

Figure S1. The median-joining network of mtDNA control region haplotypes of captive forest musk deer in Shaanxi population and Sichuan population.

Note: Blue: Shaanxi; red: Sichuan; yellow: shared; Black: missing.
