# Supplementary material for: mtDNA CR Evidence Indicates High Genetic Diversity of Captive Forest Musk Deer in Shaanxi Province, China
Source: Animals (Basel). 2023 Jul 4;13(13):2191. doi: 10.3390/ani13132191 (PMC10339889; doi:10.3390/ani13132191)
Supplement: Supplementary file 1 [file animals-13-02191-s001.zip › Table S1. The information of reagents used..pdf]

Table S1. The information of reagents used.

Reagents	Manufacturer	Location purchased
DNeasy Blood/Tissue/Cell Kit	TIANGEN	China
Phosphate Buffer Solution	Solarbio	China
Ethanol	XILONG	China
2×Es Taq MasterMix (Dye)	CWBIO	China
D 2000 DNA Marker	TIANGEN	China
GeneRed nucleic acid gel stain	TIANGEN	China
ddH2O	CWBIO	China
TAE Buffer (Premixed Powder)	Coolaber	China
Agarose	Biowest	Spain
