# Supplementary material for: mtDNA CR Evidence Indicates High Genetic Diversity of Captive Forest Musk Deer in Shaanxi Province, China
Source: Animals (Basel). 2023 Jul 4;13(13):2191. doi: 10.3390/ani13132191 (PMC10339889; doi:10.3390/ani13132191)
Supplement: Supplementary file 1 [file animals-13-02191-s001.zip › Table S2. The information of labware used..pdf]

Table S2. The information of labware used.

Labware	Manufacturer	Location purchased
Vortex Genie	Scientific Industries	America
Metal Bath	BIOER	China
Centrifuge	cence	China
High-speed Centrifuge	Sigma	Germany
Palm Centrifuge	IKA	Germany
PCR Instrument	Analytik Jena	Germany
Electron Balance	BSISL	China
Microwave oven	Galanz	China
Electrophoresis System	BEIJING LIUYI	China
Gel imager System	Bio-Rad	America
Ultra-low temperature refrigerator	Haier	China
Pipette	Eppendorf	Germany
