# Supplementary material for: mtDNA CR Evidence Indicates High Genetic Diversity of Captive Forest Musk Deer in Shaanxi Province, China
Source: Animals (Basel). 2023 Jul 4;13(13):2191. doi: 10.3390/ani13132191 (PMC10339889; doi:10.3390/ani13132191)
Supplement: Supplementary file 1 [file animals-13-02191-s001.zip › Table S3. Tajimaí»s D and Fuí»s Fs tests of captive forest musk deer populations in Shaanxi Province..pdf]

Table S3. Tajima's *D* and Fu's *Fs* tests of captive forest musk deer populations in Shaanxi Province.

Population	R	FM	PZH	HX	LD	HD	GL
Tajima's *D*	0.66418<br>(-)	0.27931<br>(-)	0.52575<br>(-)	1.48210<br>(-)	-0.64219<br>(-)	0.90019<br>(-)	-0.56156<br>(-)
Fu's *Fs*	8.16210<br>(-)	8.98281<br>(-)	-0.33736<br>(-)	16.93670<br>(-)	2.10982<br>(-)	18.46125<br>(-)	1.71356<br>(-)

Note: (-) means p-values > 0.05.
